# Comparison of Antibacterial Effects of ZnO and CuO Nanoparticles Coated Brackets against *Streptococcus Mutans*

**Published:** 2015-09

**Authors:** Baratali Ramazanzadeh, Arezoo Jahanbin, Masoud Yaghoubi, Nasser Shahtahmassbi, Kiarash Ghazvini, Mohammadtaghi Shakeri, Hooman Shafaee

**Affiliations:** 1 Dental Research Center, Dept. of Orthodontics, School of Dentistry, Mashhad University of Medical Sciences, Mashhad, Iran.; 2 Dept. of Orthodontics, School of Dentistry, Bojnourd University of Medical Sciences, Bojnord, Iran.; 3 Dept. of Physics, Ferdowsi University of Mashhad, Mashhad, Iran.; 4 Dept. of Microbiology, Ghaem Hospital, Mashhad University of Medical Sciences, Mashhad, Iran.; 5 Dept. of Community Medicine and Public Health, Ghaem Hospital, Mashhad University of Medical Sciences, Mashhad, Iran.; 6 Dental Research Center, Dept. of Orthodontics, School of Dentistry, Mashhad University of Medical Sciences, Mashhad, Iran.

**Keywords:** Nanotechnology, Bracket, Streptococcus Mutans

## Abstract

**Statement of the Problem:**

During the orthodontic treatment, microbial plaques may accumulate around the brackets and cause caries, especially in high-risk patients. Finding ways to eliminate this microbial plaque seems to be essential.

**Purpose:**

The aim of this study was to compare the antibacterial effects of nano copper oxide (CuO) and nano zinc oxide (ZnO) coated brackets against *Streptococcus mutans* (*S.mutans*) in order to decrease the risk of caries around the orthodontic brackets during the treatment.

**Materials and Method:**

Sixty brackets were coated with nanoparticles of ZnO (n=20), CuO (n=20) and CuO-ZnO (n=20). Twelve uncoated brackets constituted the control group. The brackets were bonded to the crowns of extracted premolars, sterilized and prepared for antimicrobial tests (*S.mutans* ATCC35668). The samples taken after 0, 2, 4, 6 and 24 hours were cultured on agar plates. Colonies were counted 24 hours after incubation. One-way ANOVA and Tukey tests were used for statistical analysis.

**Results:**

In CuO and CuO-ZnO coated brackets, no colony growth was seen after two hours. Between 0-6 hours, the mean colony counts were not significantly different between the ZnO and the control group (*p*>0.05). During 6-24 hours, the growth of *S.mutans* was significantly reduced by ZnO nanoparticles in comparison with the control group (*p*< 0.001). However, these bacteria were not totally eliminated.

**Conclusion:**

CuO and ZnO-CuO nanoparticles coated brackets have better antimicrobial effect on *S.mutans* than ZnO coated brackets.

## Introduction

White-spot lesions are the well-known side effect of fixed orthodontic treatment. During orthodontic treatment, increased proliferation of the facultative bacterial population, including *S.mutans*, leads to a decrease in pH which in turn can lead to development of white-spot lesions and eventually to cavitation and caries extending into the dentin.[1-3]

Recently, nanotechnology has legitimated the development of new properties of materials. The shift from microparticles to nanoparticles (<100nm in diameter) comprises an increment in relation to the surface area among other alterations in properties. A larger surface area of the nanoparticles provides more interfaces with other organic and inorganic molecules.[4]

The antimicrobial properties of silver nanoparticle, known as the most common nanoparticle, are well recognized and numerous mechanisms for their bactericidal effects have been anticipated.[5-8] Silver ions are broadly employed as bactericides in catheters, burn wound care, and in the dental practice.[9]

Although only a few studies have reported the antibacterial properties of copper and zinc nanoparticles, they have also shown copper and zinc nanoparticles to have a significant role as bactericidal agents.[4, 10-11] In this regard, Sierra *et al.* showed a higher antimicrobial effect against *S.mutans* of silver nanoparticles at lower concentrations than gold or zinc, which would permit attaining imperative clinical effects with lesser toxicity.[4] In another study, Ruparelia *et al.* demonstrated that silver and copper nanoparticles have great promise as an antimicrobial agent against *E. coli*, *Bacillus subtilis*, and *Staphylococcus aureus*. Furthermore, their microbial tests have suggested that for all cultures of *Escherichia coli (E. coli)* and *Staphylococcus aureus*, the antimicrobial action of the silver nanoparticles was superior.[11]

As there are concerns regarding the biological safety of silver nanoparticles (because of the closeness of size of nanosilver to silver ions) and due to the pigmentation effect of silver nanoparticles on teeth,[5-7] ZnO and CuO nanoparticles were used in this investigation. The purpose of this study was to examine the antimicrobial effects of coated brackets with ZnO and CuO nanoparticles against *S.mutans*.

## Materials and Method

For preparation of CuO nanoparticles, aqueous solution of copper acetate (0.02 M) was prepared in round bottom flask. Then, 1 mL glacial acetic acid was added to the above-mentioned aqueous solution and heated to 100^º ^C with constant stirring. About 0.4 g of NaOH was added to the heated solution till pH reached 6-7. A large amount of black precipitate was formed immediately. It was centrifuged and washed 3-4 times with deionized water. The obtained precipitate was dried in air for 24 hours.[12] In order to prepare ZnO nanoparticles, sodium hydroxide solution was added to the aqueous solution of zinc sulfate slowly dropwise in a ratio of 1:2 under vigorous stirring and the stirring was continued for 12 hours. The precipitate obtained was filtered and washed thoroughly with de-ionized water. The precipitate was dried in an oven at 100^º ^C and ground to fine powder.[13-14]

**Figure 1:**
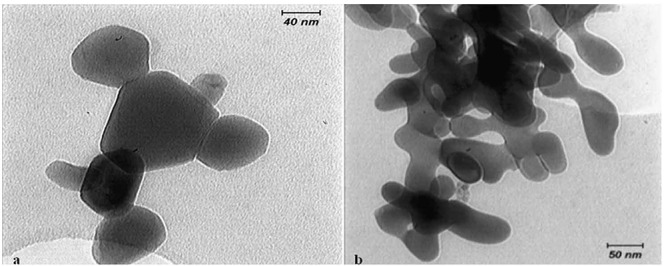
: TEM scan of ZnO nanoparticles (× 30000 magnification)  b: TEM scan of CuO nanoparticles (× 30000 magnification)

**Figure 2:**
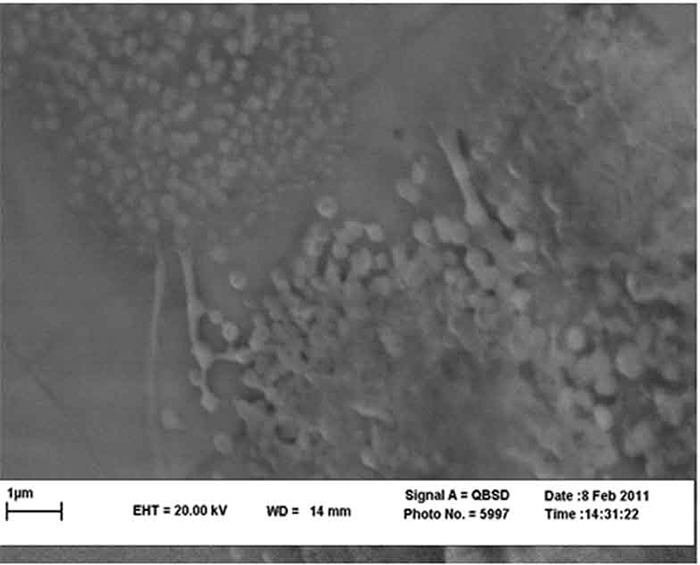
SEM scan of bracket coated with CuO-ZnO nanoparticles (× 30000 magnification)

The size and morphology of ZnO and CuO nanoparticles was examined using a transmission electron microscope (TEM; Philips CM200, Netherlands). According to the TEM evaluation, the mean diameter of ZnO and CuO nanoparticles was 45 and 37 nm, respectively (Figure 1). In this study, spray pyrolysis was used for bracket coating. The spray pyrolysis is an excellent method for deposition of thin films of metallic oxides, in which, a starting solution, containing metal precursors is sprayed by means of a nozzle, assisted by a carrier gas, over a hot substrate. When the fine droplets reach the hot substrate, the solid compounds react on the surface and a new chemical compound is developed. It should be noted that this coating was only bonded to the metal surface.[15] The coating was approved by using scanning electron microscope (SEM; Philips XL30, Netherlands) (Figure 2).

For ensuring the antimicrobial feature of nanoparticles and because of the easy deposition of nanoparticles on glass slides, CuO and ZnO thin films were deposited onto glass substrates at 400 °C by spray pyrolysis. After performing antimicrobial tests, steel pieces in the size of 2×1 cm were used for sequent deposition on the metal. The final step of sequent deposition was done on the orthodontic brackets.

This study used 72 orthodontic brackets of upper premolar teeth with 0.018 inch slots (Dentaurum; Pforzheim, Germany) that were divided into four groups. In ZnO group, the brackets were covered with ZnO nanoparticles (20 brackets). In CuO group the brackets were covered with CuO nanoparticles (20 brackets). In ZnO-CuO group, the brackets were covered with CuO and ZnO nanoparticles with 1:1 ratio (20 brackets). In the control group, the brackets were prepared without any special cover (12 brackets).

Seventy-two upper first premolars extracted for orthodontic purposes were selected and stored in distilled water at room temperature until required. No teeth had carious lesions or enamel surface defects. Their crowns were cut and their endings were blocked by flowable composite (Heliomolar; Ivoclar Vivadent AG, Principality of Liechtenstein). The buccal surface of each premolar was conditioned with phosphoric acid 35% (Dent Zar Inc; USA). A standard edgewise twin metal bracket (Dentaurum; Pforzheim, Germany) was bonded with a self-cured composite (Alfa Dent; Prime Dent, USA) to the center of the buccal surface of each tooth. Then, the teeth and brackets were sterilized by an autoclave at 120°C and a pressure of 15 psi. In this study we used the reference strain of *S.mutans* (ATCC 35668) derived from the American Type Culture Collection (ATCC; Manassas, Virginia, USA).

The standard sample of *S.mutans *was cultured and a suspension containing bacteria in a logarithmic phase with a concentration of 1.5×108 CFU.ml-1 was prepared. Subsequently, the suspension was diluted by physiologic serum to decrease the suspension concentration to 1.5×105 CFU/mL.

Each sample was placed in a separate test tube. Then 1 mL of the bacterial suspension was added to the teeth and brackets which were previously sterilized in an autoclave. Consequently, the teeth and bonded brackets were completely covered with the bacterial suspension. The samples were incubated in a shaker incubator at 37°C using 180 shakes per minute.

The suspension (10µL) was taken from each tube in intervals of 0, 2, 4, 6 and 24 hours and were cultured on the plates containing nutrient medium (Brain Heart Agar + Sheep Blood Agar). The number of colonies was counted and recorded 24 hours after incubation.

The data were analyzed by SPSS software, version 15. One-way ANOVA and Tukey tests were used for statistical analysis. 

## Results

According to Table 1, in ZnO coated brackets, one sample was omitted in each interval because of bacterial contamination. Table 1 shows that in the CuO and ZnO-CuO groups, bacterial population reduced to zero within two hours. The ZnO group, in comparison with the control group was effective in decreasing the number of *S.mutans* colonies, but had less antimicrobial effect compared with the two former groups. Mean difference in the number of colonies in the interval of 0 to 2 hours, 4 to 6 hours, and 6 to 24 hours in different groups were compared by the One-way ANOVA (Table 2). The results revealed a significant difference in the intervals of 0 to 2, 4 to 6, 6 to 24 hours; however, between 2 to 4 hours the mean difference in colony counts was not significant.

**Table 1:** The mean±SD of the counted number of colonies in various groups in intervals of 0, 2, 4, 6 and 24 hours

**Group**	**Time** **(hour)**	**Mean** **(CFU)**	**SD**	**No.** **of Samples**
ZnO	0	140.84	65.79	19
	2	96.78	46.19	19
	4	96.10	75.64	19
	6	55.89	51.95	19
	24	219.32	304.53	19
CuO	0	145.20	65.77	20
	2	8.80	22.76	20
	4	0	0	20
	6	0	0	20
	24	0	0	20
ZnO-CuO	0	134.10	69.78	20
	2	0	0	20
	4	0	0	20
	6	0	0	20
	24	0	0	20
Control	0	165.91	53.19	12
	2	139.33	43.71	12
	4	135.08	68.93	12
	6	117.75	66.68	12
	24	921.25	823.68	12

**Table 2:** Comparison of the change in the number of colonies in the studied groups in different intervals

**Variation in colony count in various intervals between groups**	**DF**	**P-value**
0-2 hours	3	0.000*
2-4 hours	3	0.940
4-6 hours	3	0.001*
6-24 hours	3	0.000*

*Significant *p*< 0.05

The Tukey test showed that the mean differences were statistically significant among all the study groups except in ZnO-CuO group (*p*= 0.999), as well as in the ZnO and control groups (*p*=0.844) with an interval of 0 to 2 hours (Table 3). As illustrated in Table 4, the anti-microbial effect of the ZnO group in the interval of 6 to 24 hours showed a statistically significant difference (*p*<0.001). Based on the Tukey test in the paired comparison of groups in the interval of 0 to 24 hours, all of the groups were significantly different (*p*<0.05) except the CuO and ZnO-CuO groups (*p*= 0.998). In the ZnO, CuO and ZnO-CuO groups, the number of colonies in different intervals was lower than the control group. (Table 5)

**Table 3:** Comparison of groups two by two in the interval of 0 to 2 hours

**Group I**	**Group J**	**Mean Difference (I-J)**	**Standard Error**	**P-value**	**Confidence Interval**	
					**Lower Bound**	**Upper Bound**
ZnO	CuO	-92.40	18.48	0.000*	-141.13	-43.66
	ZnO-CuO	-90.10	18.48	0.000*	-138.83	-41.36
	Control	17.41	21.20	0.844	-38.48	73.31
CuO	ZnO-CuO	2.30	17.99	0.999	-45.13	49.73
	Control	109.81	20.77	0.000*	55.04	164.58
ZnO-CuO	Control	107.51	20.77	0.000*	52.74	162.28

*Significant *p*< 0.001

**Table 4:** Comparison of change in the number of colonies in different intervals between ZnO and the control group

**Time (hours)**	**Mean difference (I-J)**	**Standard Error**	**P-value**	**Confidence Interval**	
				**Lower Bound**	**Upper Bound**
2-4	-1.08	16.59	1.000	-44.82	42.66
4-6	20.16	11.76	0.325	-10.83	51.17
6-24	643.76	1.36	0.000*	283.74	1003.77

*Significant *p*< 0.001

**Table 5:** Paired comparison of groups based on the overall average of the number of colonies in the interval of 0 to 24 hours

**Group I**	**Group II**	**Mean difference (I-II)**	**Standard Error**	**P-value**	**Confidence Interval**	
					**Lower Bound**	**Upper Bound**
ZnO	CuO	90.98	24.42	0.001*	27.91	154.06
	ZnO-CuO	94.96	24.42	0.001*	31.89	158.04
	Control	-174.07	28.11	0.001*	-246.67	-101.43
CuO	ZnO-CuO	3.98	24.11	0.998	-58.27	66.23
	Control	-265.06	27.84	0.000*	-336.95	-193.17
ZnO-CuO	Control	-269.04	27.84	0.000*	-340.93	-197.15

*Significant    *p*< 0.05

## Discussion

Nanotechnology has opened new trends in health. One of its most interesting characteristics is the antimicrobial feature that some nanoparticles demonstrate.[4-5,7]

According to Table 1, the best findings were observed in group CuO and ZnO-CuO, which reduced the bacterial population to zero within two hours. The ZnO group, in comparison with the control group, was also effective in decreasing the number of *S.mutans* colonies but had less antimicrobial effects compared with the two former groups.

In the ZnO and control groups, we observed a decrease in the number of bacteria between 0 to 6 hours (Table 2). This may be due to the reduction of nutrients as a result of the dilution of bacterial suspension; bacterial growth was not observed in the lag phase and as they continued to die naturally, the population of microorganisms decreased as time passed.[4-5]

Between 6 to 24 hours, an increase in the bacterial population was observed in the control, as well as in ZnO groups, which may be related to the long interval of 18 hours between the two colony counts. However, the rate of bacterial growth was lower in the ZnO group because of its bacteriostatic characteristics. This may be due to the fact that the microorganisms had adapted to a new environment and their entrance into the logarithmic growth phase was probable.

Many studies examined the effects of different nanoparticles on various bacterial strains.[11, 18-20] One of the important mechanisms of the antimicrobial effects of nanoparticles is the destruction of the microorganism membrane.[5, 16-17] As *Bacillus subtilis* and *S. mutans* are both gram-positive bacteria, it may be expected that Cu nanoparticles may also affect* S. mutans*, as their anti-microbial effects on *S. mutans* were observed in our study.

In the study by Ruparelia *et al.*, the antimicrobial effect of Cu and silver nanoparticles against different types of bacteria was investigated and they concluded that bacterial sensitivity to nano particles varied depending on microbial species.[11] In the study by Yoon *et al.*, Cu nanoparticles showed favorable anti-microbial effects on *E-coli* and *Bacillus subtilis*, which is in agreement with the results obtained by Ruparelia *et al.*[18] In the study conducted by Adams *et al.*, the antibacterial effect of ZnO nanoparticles on *Bacillus subtilis* and *E-coli* was showed.[19]

Another study showed that ZnO-Parylene-glass composites had a significant antibacterial activity against *E-coli* (gram-negative) and *Staphylococcus aureus* (gram-positive) strains.[20] In this regard, few studies have been published on the antimicrobial feature of CuO and ZnO nanoparticles against *S. mutans*.[4]

In the study performed by Hernández-Sierra *et al.*, the antimicrobial effect of ZnO nanoparticles was in agreement with our findings. In their study, the colloidal nanoparticles were studied but in our study sequent deposition of nanoparticles on the bracket surfaces were scrutinized.[4] There was an unfavorable color change in brackets after heating at high temperatures. Considering that ZnO nanoparticles are transparent, it was hard to differentiate them from uncoated glasses when they were deposited on glass. However, when the brackets were deposited with ZnO nanoparticles at 400°C, the color of the bracket changed briefly to very light gold, which was just as beautiful as steel brackets.

**Figure 3:**
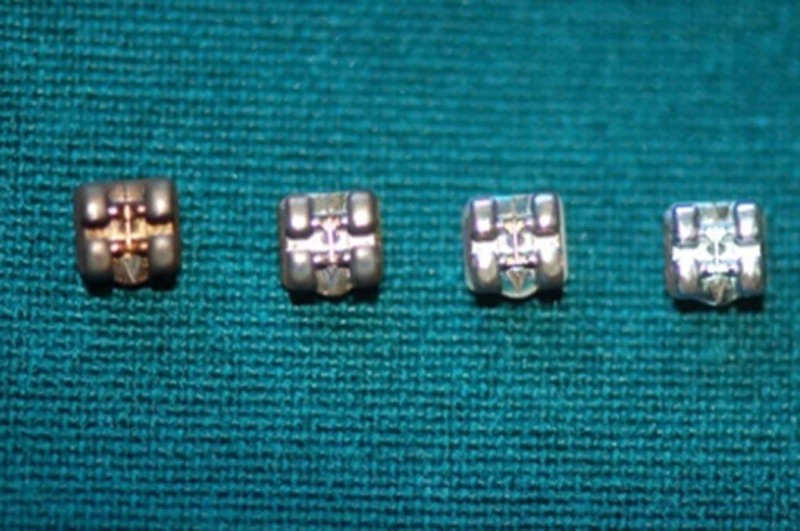
Brackets color changes after coating with CuO-ZnO nanoparticles, CuO nanoparticles and ZnO nanoparticles in comparison with controls (left to right).

In the CuO and ZnO-CuO groups, the color of the brackets changed to copper, which was cosmetically unsuitable (Figure 3).

Considering that fact that brackets in the mouth are in contact with saliva, abrasions caused by a tooth-brush and toothpaste and different types of foods and drinks with various temperatures and pHs, the authors suggest to evaluate the stability of the coated layers in the future studies.

Although the findings of this study on the antimicrobial effect of brackets in short-term were positive, other investigations should be carried out in order to prove our notion. 

## Conclusion

In the short term, the antimicrobial effect of coated brackets with CuO and ZnO- CuO nanoparticles on *S.mutans* was excellent since after almost two hours the number of bacteria were reduced to zero. The coated brackets with ZnO nanoparticles ranked second due to its antimicrobial effects which, although in comparison with the control group caused dramatic reduction in the number of *mutans*, it could not reduce the population of *S. mutans* to zero even after 24 hours.

## References

[1] Featherstone JD (2003). The caries balance: contributing factors and early detection. J Calif Dent Assoc.

[2] Featherstone JD, Domejean-Orliaguet S, Jenson L, Wolff M, Young DA (2007). Caries risk assessment in practice for age 6 through adult. J Calif Dent Assoc.

[3] Richter AE, Arruda AO, Peters MC, Sohn W (2011). Incidence of caries lesions among patients treated with comprehensive orthodontics. Am J Orthod Dentofacial Orthop.

[4] Hernández-Sierra JF, Ruiz F, Pena DC, Martínez-Gutiérrez F, Martínez AE, Guillén Ade (2008). The antimicrobial sensitivity of Streptococcus mutans to nanoparticles of silver, zinc oxide, and gold. Nanomedicine.

[5] Sondi I, Salopek-Sondi B (2004). Silver nanoparticles as antimicrobial agent: a case study on E. coli as a model for Gram-negativebacteria. J Colloid Interface Sci.

[6] Siva Kumar V, Nagaraja BM, Shashikala V, Padmasri AH, Madhavendra SS, Raju BD (2004). Highly efficient Ag/C catalyst prepared by electro-chemical deposition method in controlling microorganisms in water. J Mol Catal A Chem.

[7] Jain P, Pradeep T (2005). Potential of silver nanoparticle-coated polyurethane foam as an antibacterial water filter. Biotechnol Bioeng.

[8] Cho K, Park J, Osaka T, Park S (2005). The study of antimicrobial activity andpreservative effects of nanosilver ingredient. Electrochim Acta.

[9] Kim JS, Kuk E, Yu KN, Kim JH, Park SJ, Lee HJ (2007). Antimicrobial effects of silver nanoparticles. Nano-medicine.

[10] Cioffi N, Torsi L, Ditaranto N, Tantillo G, Ghibelli L, Sabbatini L (2005). Copper nanoparticle/polymer composites with antifungal and bacteriostatic properties. Chem Mater.

[11] Ruparelia JP, Chatterjee AK, Duttagupta SP, Mukherji S (2008). Strain specificity in antimicrobial activity of silver and copper nanoparticles. Acta Biomater.

[12] Lanje AS, Sharma SJ, Pode RB, Ningthoujam RS (2010). Synthesis and optical characterization of copper oxide nanoparticles. Adv Appl Sci Res.

[13] Daneshvar N, Aber S, Seyed Dorraji MS, Khataee AR, Rasoulifard MH (2008). Preparation and Investigation of Photocatalytic Properties of ZnO Nanocrystals: Effect of Operational Parameters and Kinetic Study. Int J Chem Biol Eng.

[14] Kumar SS, Venkateswarlu P, Rao VR, Rao GN (2013). Synthesis, characterization and optical properties of zinc oxide nanoparticles. Int. Nano Lett.

[15] Okuyama K, Wuled Lenggoro I (2003). Preparation of nanoparticles via spray route. Chemical engineering science.

[16] Gorelick L, Geiger AM, Gwinnett AJ (1982). Incidence of white spot formation after bonding and banding. Am J Orthod.

[17] Gogoi SK, Gopinath P, Paul A, Ramesh A, Ghosh SS, Chattopadhyay A (2006). Green fluorescent protein-expressing Escherichia coli as a model system for investigating theantimicrobial activities of silver nanoparticles. Langmuir.

[18] Yoon KY, Hoon Byeon J, Park JH, Hwang J (2007). Susceptibility constants of Escherichia coli and Bacillus subtilis to silver and copper nanoparticles. Sci Total Environ.

[19] Adams LK, Lyon DY, Alvarez PJ (2006). Comparative eco-toxicity of nanoscale TiO2, SiO2, and ZnO water suspensions. Water Res.

[20] Applerot G, Abu-Mukh R, Irzh A, Charmet J, Keppner H, Laux E (2010). Decorating parylenecoated glass with ZnO nanoparticles for antibacterial applications: acomparative study of sonochemical, microwave, and microwave-plasma coating routes. ACS Appl Mater Interfaces.

